# Differential Effects of Cancer-Associated Mutations Enriched in Helix H3 of PPARγ

**DOI:** 10.3390/cancers12123580

**Published:** 2020-11-30

**Authors:** Dong Man Jang, Jun Young Jang, Hyun-Jung Kim, Byung Woo Han

**Affiliations:** 1 Research Institute of Pharmaceutical Sciences, College of Pharmacy, Seoul National University, Seoul 08826, Korea; jdm721@snu.ac.kr (D.M.J.); nosvc4@snu.ac.kr (J.Y.J.); 2 College of Pharmacy, Chung-Ang University, Seoul 06974, Korea; hyunjungkim@cau.ac.kr

**Keywords:** cancer mutation, helix H3, ligand-binding domain, PPARγ, tumor microenvironment

## Abstract

**Simple Summary:**

The high frequency of mutations in helix H3 of the peroxisome proliferator-activated receptor gamma (PPARγ) ligand-binding domain (LBD) in various cancers suggest that this region has an important role in tumorigenesis. In this study, we performed bioinformatics, structural and biochemical analyses to characterize PPARγ LBDs with helix H3 mutations found in cancers. In the absence of ligands, PPARγ Q286E that was a mutation found in patients with bladder cancer induced a constitutively active conformation of PPARγ and promoted coactivator recruitment, providing evidence for tumorigenic effects. A number of other mutations reduced PPARγ activation by various mechanisms. Accordingly, mutations in helix H3 of PPARγ LBD have a wide range of effects and are key candidates for the development of biomarkers and targeted therapies.

**Abstract:**

Peroxisome proliferator-activated receptor gamma (PPARγ) has recently been revealed to regulate tumor microenvironments. In particular, genetic alterations of PPARγ found in various cancers have been reported to play important roles in tumorigenesis by affecting PPARγ transactivation. In this study, we found that helix H3 of the PPARγ ligand-binding domain (LBD) has a number of sites that are mutated in cancers. To uncover underlying molecular mechanisms between helix H3 mutations and tumorigenesis, we performed structure‒function studies on the PPARγ LBDs containing helix H3 mutations found in cancers. Interestingly, PPARγ Q286E found in bladder cancer induces a constitutively active conformation of PPARγ LBD and thus abnormal activation of PPARγ/RXRα pathway, which suggests tumorigenic roles of PPARγ in bladder cancer. In contrast, other helix H3 mutations found in various cancers impair ligand binding essential for transcriptional activity of PPARγ. These data indicate that cancer-associated mutations clustered in helix H3 of PPARγ LBD exhibit differential effects in PPARγ-mediated tumorigenesis and provide a basis for the development of new biomarkers targeting tumor microenvironments.

## 1. Introduction

Peroxisome proliferator-activated receptor gamma (PPARγ) belonging to thyroid hormone receptor-like nuclear receptor subfamily 1 is a ligand-inducible transcription factor [1]. PPARγ forms a heterodimer with retinoid X receptor α (RXRα), recruits various co-regulators and regulates the expression of downstream target proteins [2]. The transcriptional regulation of PPARγ plays an important role in adipogenesis, insulin sensitization, lipid metabolism, glucose homeostasis and inflammation [3,4]. Thus, PPARγ is a target for the treatment of metabolic diseases, including type 2 diabetes, obesity and atherosclerosis [5]. In fact, PPARγ full agonist thiazolidinedione (TZD) drugs, such as pioglitazone and rosiglitazone, have been widely used for the treatment of diabetes, despite known side effects, such as weight gain, fluid retention and bone loss [6,7,8].

Recently, PPARγ has emerged as a mediator of tumor microenvironments [9,10]. There is accumulating evidence for tumorigenic roles of PPARγ in a variety of cancers, including colon, breast, lung, prostate and bladder cancers [5,11]. In general, activation of PPARγ by agonists exerts inhibitory effects on tumor growth by various mechanisms, including apoptosis, cell cycle arrest and inhibitions of angiogenesis or differentiation [12,13,14,15]. For example, PPARγ activation has been known to inhibit the expression of angiogenesis-associated proteins, such as basic fibroblast growth factor (bFGF) and vascular endothelial growth factor (VEGF) [9,16,17]. Moreover, TZD drugs elicit tumor-suppressive effects to induce apoptosis and inhibit cell proliferation in various cancer cells by regulating apoptosis-associated proteins, such as cyclooxygenase-2 (COX-2), B-cell lymphoma 2 (Bcl-2), epidermal growth factor receptor (EGFR) and Fas ligand (FasL) [18,19,20,21,22]. In a recent study, lung cancer cells treated with PPARγ agonists remarkably lost migratory and invasive properties essential for cancer metastasis [10]. Furthermore, there have been clinical trials for various cancers using PPARγ agonists such as pioglitazone [23].

In addition to the pharmacological regulation of PPARγ in cancers, *PPARG* genetic alterations resulting in a loss- or gain-of-function have been reported in a variety of cancer types [24,25]. Due to the tumor-suppressive effect of PPARγ, some loss-of-function mutations in *PPARG* have been regarded as tumorigenic factors [26,27]. Indeed, somatic *PPARG* loss-of-function mutations decrease the transcriptional activity of PPARγ by inhibiting ligand binding in sporadic colon cancer [24]. In addition, a *PPARG* germline mutation found in a patient with colorectal cancer impairs the transactivation potential and impedes agonist positioning [28]. On the other hand, gain-of-function mutations in *PPARG* have also been found in cancers [25,29]. Some recurrent *PPARG* mutations in luminal bladder cancer increase the ligand-independent transcriptional activity of PPARγ, resulting in the upregulation of PPARγ target genes [25,29]. The activation of the PPARγ/RXRα pathway as a result of gain-of-function mutations impairs CD8^+^ T-cell infiltration by inhibiting the expression and secretion of inflammatory cytokines and confers acquired resistance to immunotherapies [30]. Owing to the ongoing discovery of *PPARG* mutations in various cancers, the molecular basis of PPARγ mutations found in cancers would be an emerging topic of great importance.

Helix H3 of the PPARγ ligand-binding domain (LBD) is essential for ligand binding and PPARγ activation and harbors a relatively large number of sites that are mutated in cancers. In this study, to identify the functional relationship between mutations in helix H3 of PPARγ LBD and cancer development or progression, structural and biochemical analyses were performed using crystal structures and in vitro assays for RXRα dimerization, coactivator recruitment and transcriptional activity. In addition, we measured binding affinities between PPARγ LBD mutants and endogenous ligands. Our results reveal differential effects of the helix H3 mutations in PPARγ transcriptional activity and provide a molecular mechanism underlying PPARγ-mediated tumorigenesis.

## 2. Results

### 2.1. Structural Comparison of PPARγ LBDs Containing Helix H3 Mutations Found in Various Cancers

**Figure 1:**
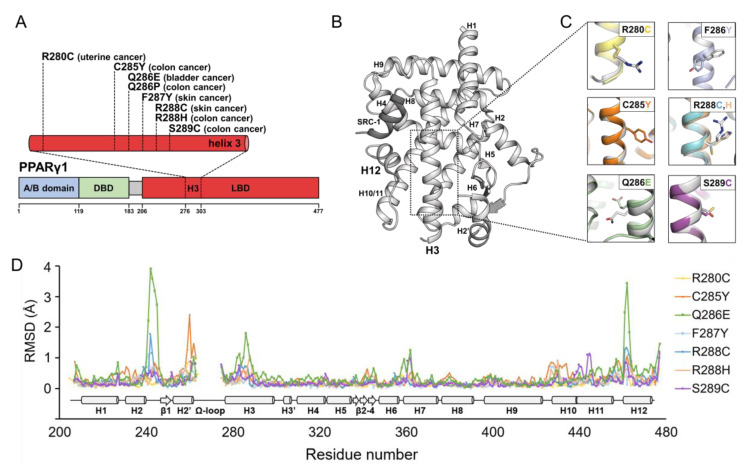
The helix H3 mutations of PPARγ ligand-binding domain (LBD) found in cancers and their crystal structures. (**A**) Schematic diagrams displaying domain compositions and mutations in helix H3 of PPARγ LBD. Sequences are numbered according to the PPARγ1 isoform. A/B domain, DBD and LBD indicate N-terminal domain, DNA-binding domain and ligand-binding domain, respectively. (**B**) Overall structure of PPARγ LBD WT displayed as a cartoon representation colored in light grey. A SRC-1 peptide is colored in dark grey. (**C**) The structural comparisons of apo PPARγ LBD WT and mutants. The structure of PPARγ LBD WT is superposed with those of PPARγ LBD mutants. The side chains of the WT and mutated residues are shown as stick representations, in light grey color for the WT and various colors for the mutants. (**D**) The comparison of C_α_ RMSD values for the PPARγ LBD mutant structures against the PPARγ LBD WT structure. The mutant structures of PPARγ LBD R280C, C285Y, Q286E, F287Y, R288C, R288H and S289C are compared to the PPARγ LBD WT structure in the presence of a SRC-1 peptide (PDB ID: 6JQ7). Secondary structure elements are displayed along residue numbers.

Using the cBioPortal and COSMIC servers [31,32,33], we obtained 45,665 cancer samples from publicly available databases, such as The Cancer Genome Atlas (TCGA) and International Cancer Genome Consortium (ICGC). Among 282 mutation samples in PPARγ, we focused on 189 missense mutations. Interestingly, we found that helix H3 of PPARγ LBD is enriched for mutations that are found in cancers; helix H3 accounts for only 9.4% of the amino acids in the PPARγ LBD but 19.6% of PPARγ LBD mutations found in cancers (19 mutations in helix H3 among 97 mutations distributed in the PPARγ LBD). Because it might provide insight into the mechanisms underlying PPARγ-mediated tumorigenesis, we focused on mutations in helix H3 of PPARγ LBD for further structure–function analyses. In addition to the mutation data from bioinformatics analyses, we added two helix H3 mutations of apo PPARγ LBD (Q286P and S289C) reported in literatures [24,28]. We determined the crystal structures of PPARγ LBDs containing helix H3 mutations found in cancer patients at 1.9 Å to 2.7 Å resolution with or without an SRC-1 peptide, which are monomeric or dimeric in an asymmetric unit, respectively (Table S1). The mutations in helix H3 of PPARγ LBD included C285Y, Q286P, R288H and S289C identified in patients with colorectal cancer, F287Y, R288C from patients with skin cancer, Q286E from a patient with bladder cancer and R280C from a patient with uterine cancer (Figure 1A and Table S2). The structures of PPARγ LBD wild-type (WT) and mutants adopted a general nuclear receptor fold comprising one bundle of 13 α-helices and one four-stranded β-sheet (Figure 1B). In the mutant structures, the mutant residues were well-modeled (Figure 1C and Figure S1). The overall structures of apo PPARγ LBD WT and mutants were nearly identical with root-mean-square deviation (RMSD) values of 0.20 Å (R280C), 0.43 Å (C285Y), 0.65 Å (Q286E), 0.27 Å (F287Y), 0.31 Å (R288C), 0.29 Å (R288H) and 0.29 Å (S289C) over 260 C_α_ atom pairs of chain A (Figure 1D and Figure S2). However, noticeable structural differences in the mutants were observed in the H2–β1 loop, Ω-loop (a loop between H2′ and H3), helix H3 and H11–H12 loop with relatively high C_α_ RMSD values (Figure 1D). In particular, the Ω-loop was the most flexible region in the structure of PPARγ LBD; hence, it was only partially modeled in some of the mutant structures (Figure 1D). Since the PPARγ LBD Q286P mutant could not be crystallized, we analyzed secondary structural elements by circular dichroism (CD) experiments. Based on the CD spectra of PPARγ LBD WT, Q286P and Q286E, the overall structure of PPARγ LBD Q286P would resemble those of PPARγ LBD WT and Q286E (Figure S3).

### 2.2. PPARγ LBD Q286E Induces a Constitutively Active Conformation of PPARγ LBD

**Figure 2:**
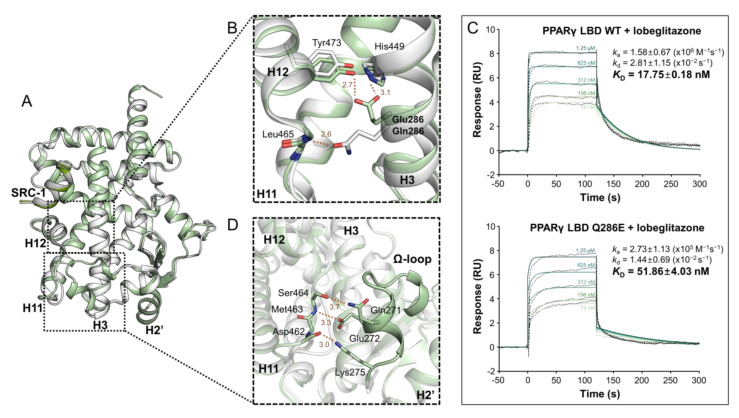
Overall structures of PPARγ LBD WT and Q286E. (**A**) Superposition of apo PPARγ LBD WT (white) and Q286E (pale green) structures. Overall structures are displayed as cartoon representations. (**B**) The region of helices H3, H11 and H12 is magnified and depicted by a black-dashed box. Stick representations are shown for the backbone of Leu465 and the side chains of Tyr473, His449, Glu286 of Q286E and Gln286 of WT. (**C**) Surface plasmon resonance (SPR) analyses of binding affinities for lobeglitazone of PPARγ LBD WT (upper) and Q286E (bottom). SPR sensorgrams show the binding of lobeglitazone at increasing concentrations to immobilized proteins of PPARγ LBD WT and Q286E. *K_D_* values shown on the right side are calculated from fitting (colored lines) onto the responses (dotted lines in black color). The data of *k*_a_, *k*_d_ and *K*_D_ are presented as the mean ± SD of two independent experiments. (**D**) The region of Ω-loop is magnified and depicted by a black-dashed box. Stick representations are shown for the side chains of Gln271, Glu272, Lys275 and Ser464 and the backbones of Asp462 and Met463 in the PPARγ Q286E structure. Hydrogen bonds are depicted by orange dashed lines and labeled with distances in Å. Oxygen and nitrogen atoms are colored in red and blue, respectively.

Among the crystal structures of 7 PPARγ LBD mutants, the structure of PPARγ LBD Q286E exhibited the highest C_α_ RMSD value, which was mainly caused by the H2′–H3 and H11–H12 regions (Figure 1D). Accordingly, we intensively compared the structures of PPARγ LBD Q286E and WT (Figure 2A). To our surprise, the mutated Glu286 residue on helix H3 directly stabilized helix H12 and induced a constitutively active form of PPARγ (Figure 2B). In particular, the O_ε_ atom of Glu286 formed hydrogen bonds with the O_η_ atom of Tyr473 in helix H12 and the N_ε_ atom of His449 in helix H11 with distances of 2.7 Å and 3.1 Å, respectively, unlike the WT structure with an interaction between Gln286 and Leu465 in the H11–H12 loop (Figure 2B). The interaction network inducing the constitutively active form of PPARγ was observed in the PPARγ Q286E mutant in the presence and absence of an SRC-1 peptide (Figure 2A and Figure S4). In addition, to examine whether the structural conformation of Glu286 interacting with Tyr473 in helix H12 exists in solution, we implemented a surface plasmon resonance (SPR) experiment using a synthetic ligand, lobeglitazone known as a full agonist interacting with Tyr473 [34]. In the SPR analysis, the binding affinity of lobeglitazone for PPARγ LBD was lower in the Q286E mutant (*K*_D_ = 52 nM) than in PPARγ WT (*K*_D_ = 18 nM) (Figure 2C). The inhibitory effect of the Q286E mutation on lobeglitazone binding to PPARγ implies that the constitutively active conformation of PPARγ Q286E upon the interaction between Glu286 and Tyr473 is physiologically relevant.

Another remarkable structural feature of the PPARγ LBD Q286E mutant is the well-defined Ω-loop with a helical structure, considering that the Ω-loop in the structures of PPARγ LBD WT and other mutants could not be modeled owing to its high flexibility (Figure 2D). The Ω-loop of the PPARγ LBD Q286E structure was stabilized by creating an extensive interaction network with an H11–H12 loop via hydrogen bonds (Figure 2D). In particular, Gln271, Glu272 and Lys275 on the Ω-loop establish hydrogen bonds with the side chain of Ser464 and backbones of Met463 and Asp462 with distances of 3.3 Å, 3.0 Å and 3.0 Å, respectively (Figure 2D). Moreover, the side chain of Glu272 on the Ω-loop formed a hydrogen bond with the side chain of Gln283 on helix H3 with a distance of 2.9 Å (Figure S5). The stabilization of Ω-loop was also observed in the structure of PPARγ LBD Q286E chain B in the absence of an SRC-1 peptide (Figure S6). Taken together, the interaction network among the Ω-loop, the H11–H12 loop and helix H3 induce structural stabilization in PPARγ Q286E, resulting in the constitutively active conformation.

### 2.3. PPARγ Q286E Recruits RXRα and MED1 with High Affinities

**Figure 3:**
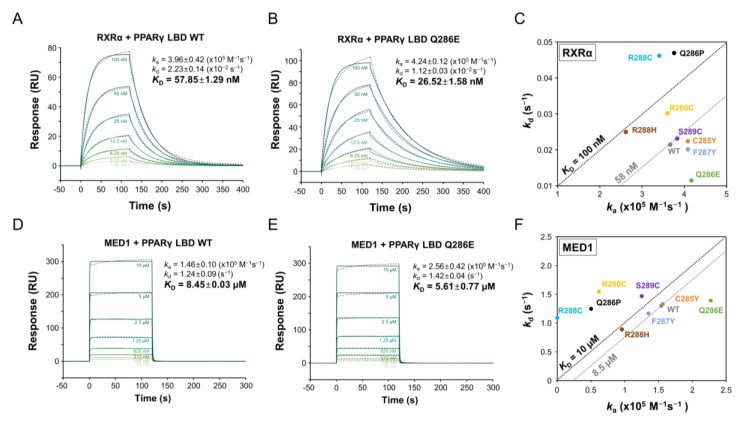
Binding affinities of PPARγ LBD WT and mutants for RXRα and MED1. (**A**,**B**) SPR sensorgrams for interactions of RXRα LBD with PPARγ LBD WT (**A**) and Q286E (**B**). SPR sensorgrams show the binding of PPARγ LBD proteins at increasing concentrations to immobilized RXRα LBD. (**D**,**E**) Sensorgrams for interactions of MED1 peptide with PPARγ LBD WT (**D**) and Q286E (**E**). SPR sensorgrams show the binding of PPARγ LBD proteins at increasing concentrations to immobilized MED1 peptides. The equilibrium dissociation constant (*K*_D_) shown on the right side is calculated by fitting (colored lines) the responses (dotted lines in black color). The data of *k*_a_, *k*_d_ and *K*_D_ are presented as the mean ± SD of two independent experiments. (**C**,**F**) Summary of *K*_D_ values for PPARγ LBD with RXRα LBD (**C**) and MED1 peptides (**F**). A *K*_D_ value is obtained from dividing the dissociation rate (*k*_d_) of Y-axis by the association rate (*k*_a_) of X-axis. The black- and grey-dashed diagonal lines represent a *K*_D_ value for a standard and a *K*_D_ value for the WT, respectively.

The transcriptional activity of PPARγ is regulated by heterodimer formation with RXRα and the recruitment of co-regulators [2]. To evaluate the effect of mutations on transcriptional activity, we quantified heterodimerization between PPARγ LBD and RXRα LBD by SPR (Figure 3A–C and Figure S7). After RXRα LBD was immobilized on the CM5 sensor chip, PPARγ LBD WT and mutant proteins were injected over the chip at concentrations ranging from 1.56 nM to 100 nM, respectively. The equilibrium dissociation constant (*K*_D_) of PPARγ LBD WT for RXRα LBD was 57.85 nM, based on a *k*_a_ value of 3.96 × 10^5^ M^−1^s^−1^ and *k*_d_ value of 2.23 × 10^−2^ s^−1^ (Figure 3A). To our surprise, PPARγ LBD Q286E exhibited a two-fold higher binding affinity for RXRα LBD with a *K*_D_ value of 26.52 nM, even though Glu286 was not located on the heterodimeric interface (Figure 3B). Interestingly, the high binding affinity of PPARγ LBD Q286E for RXRα LBD could be explained by the slow dissociation rate rather than by a fast association rate (Figure 3B). On the other hand, the PPARγ LBD R280C, C285Y, F287Y, R288H and S289C mutants exhibited similar binding affinities for RXRα LBD to that of PPARγ LBD WT, while the PPARγ LBD Q286P and R288C mutants exhibited lower binding affinities, with *K*_D_ values of 122.4 nM and 136.9 nM, respectively (Figure 3C).

Next, we evaluated the interaction between PPARγ LBD and MED1 co-activator peptides containing the LXXLL motif, where X represents any amino acid residue, by SPR (Figure 3D–F and Figure S8). PPARγ LBD WT and mutants at concentrations of 78 nM to 10 μM were injected over the MED1 peptide-immobilized CM5 chip. The equilibrium dissociation constant (*K*_D_) of PPARγ LBD WT for the MED1 peptide was 8.45 μM, calculated from a *k*_a_ value of 1.46 × 10^5^ M^−1^s^−1^ and *k*_d_ value of 1.24 s^−1^ (Figure 3D). The PPARγ LBD Q286E mutant exhibited a slightly higher binding affinity for the MED1 peptide (K_D_ = 5.61 μM) than that of PPARγ LBD WT (Figure 3E), while the other mutants exhibited similar or lower binding affinities for the MED1 peptide (Figure 3F). These results demonstrate that the PPARγ Q286E mutation favors the recruitment of RXRα and MED1, which are essential for transcriptional activity, unlike the other mutants.

### 2.4. PPARγ Q286E Exhibits Higher Transcriptional Activity than That of PPARγ LBD WT

**Figure 4:**
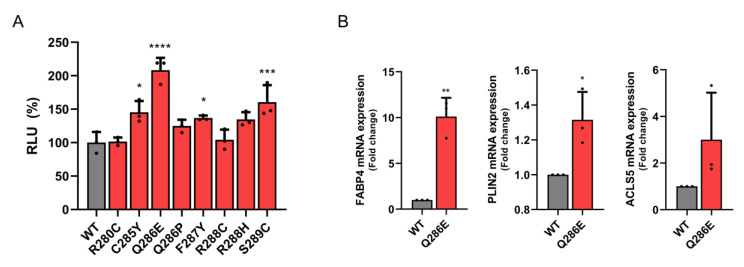
Transcriptional activity of PPARγ WT and mutants. (**A**) An expression vector encoding PPARγ full length WT or mutants (R280C, C285Y, Q286E, Q286P, F287Y, R288C, R288H and S289C) and a firefly luciferase reporter vector fused with three copies of PPARγ response element (PPRE) sequence are co-expressed in HEK293T cells with a normalization vector containing a *Renilla* luciferase gene. The firefly luciferase signals normalized by *Renilla* luciferase signals are indicated by relative luminescence unit (RLU, %) to PPARγ WT. The data are presented as the mean ± standard deviation (SD) of three independent experiments. The results for each mutant are compared to that for the WT in Dunnett’s multiple comparison test, * 0.01 < *p* < 0.05; *** 0.0001 < *p* < 0.001; **** *p* < 0.0001. (**B**) The effects of PPARγ WT and Q286E expressions on three PPARγ target genes (*FABP4*, *PLIN2* and *ACLS5*) are measured by RT-qPCR. The expression of PPARγ target genes is normalized against the basal expression of human 60S ribosomal protein *L*32. The data are presented as the mean ± SD of three independent experiments. The result for the Q286E is compared with that for the WT in Student’s *t*-test, * 0.01 < *p* < 0.05; ** *p* < 0.01.

To further elucidate correlations between biophysical and biochemical characteristics described above and functional activity, we conducted cell-based luciferase assays to measure the transcriptional activity of PPARγ. Transcriptional activities of full-length PPARγ WT and mutants in HEK293T cells were measured using a luciferase reporter vector fused to three copies of the PPARγ response element (PPRE) sequence and a normalization vector containing a Renilla luciferase gene. The transcriptional activity of PPARγ Q286E was significantly higher than that of PPARγ WT (Figure 4A). The PPARγ C285Y, F287Y and S289C mutants showed slightly higher transcriptional activities than that of PPARγ WT, whereas the R280C, Q286P and R288C mutations did not alter transcriptional activity (Figure 4A).

To further investigate how the PPARγ Q286E mutation affects the expression of PPARγ target genes, mRNA expression levels of fatty acid binding protein 4 (FABP4), long-chain fatty acid CoA ligase 5 (ACSL5) and perilipin 2 (PLIN2) were measured by RT-qPCR (Figure 4B). The PPARγ Q286E mutation resulted in a significant increase in FABP4 expression and slight increases in the levels of ACLS5 and PLIN2 (Figure 4B). Along with our biophysical and biochemical analyses, the cell-based assay clearly demonstrated that the PPARγ Q286E mutation constitutively increases the transcriptional activity of PPARγ via the stabilization of the active conformation and by promoting the recruitment of RXRα and co-activators.

### 2.5. The Activation of PPARγ Q286E Would Be Independent of Endogenous Ligands

**Figure 5:**
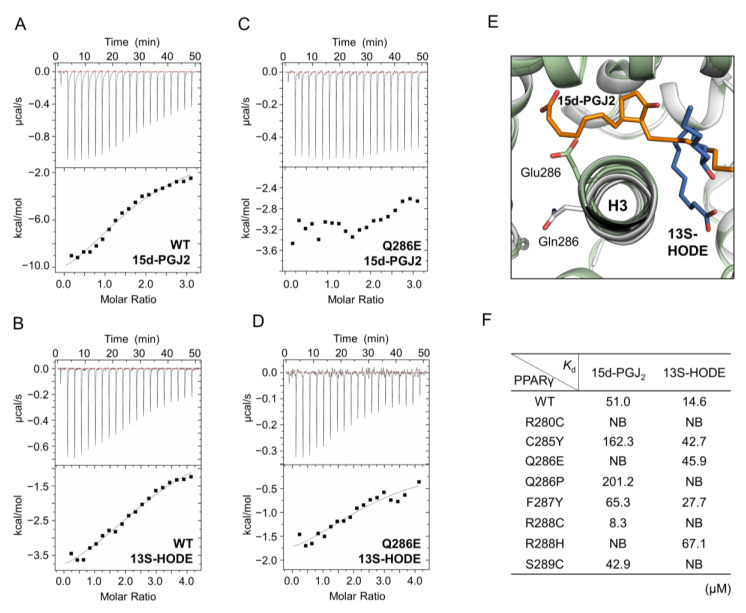
The binding affinity of PPARγ LBD for endogenous ligands. (**A**–**D**) ITC profiles for PPARγ LBD WT and Q286E with 15d-PGJ2 (**A**,**C**) and 13S-HODE (**B**,**D**). ITC analysis is conducted by titrating the endogenous ligands at a concentration of 0.9 mM into PPARγ LBD proteins at concentrations ranging from 45 μM to 60 μM. (**E**) Superposition of PPARγ LBD WT and Q286E structures with endogenous ligands of 15d-PGJ2 and 13S-HODE. The structures of PPARγ LBD WT and Q286E are shown as cartoon representations colored in white and pale green, respectively. The residues, Gln286 (white) and Glu286 (pale green) and the endogenous ligands, 15d-PGJ2 (orange) and HODE (blue), are displayed as stick representations. The ligands from the ligand-bound PPARγ LBD structures (PDB ID: 2ZK1, 2VST) are superposed onto the PPARγ LBD WT and Q286E structures. (**F**) *K*_D_ values are calculated from the fittings of the integrated molar heat upon added ligands and listed in table for PPARγ LBD WT and mutants.

Since PPARγ is physiologically regulated by endogenous ligands, it is possible that the activation of PPARγ mutants would be induced upon binding to known endogenous PPARγ ligands. Thus, we implemented isothermal titration calorimetry (ITC) experiments to measure the binding affinities of PPARγ LBD WT and mutants for 15-deoxy-∆^12,14^-prostaglandin J_2_ (15d-PGJ2) and 13*S*-hydroxy-9Z,11E-octadecadienoic acid (13*S*-HODE), which are endogenous ligands with PPARγ agonism [35,36]. In the ITC experiments, the ligands at a concentration of 0.9 mM were titrated into PPARγ LBD at concentrations ranging from 45 μM to 60 μM using 19 injections of 2 μL. PPARγ LBD WT exhibited *K*_D_ values of 51.0 μM and 14.6 μM for 15d-PGJ2 and 13S-HODE, respectively (Figure 5A,B). However, the binding affinity of PPARγ LBD Q286E for 15d-PGJ2 was not detected and for 13S-HODE was lower (*K*_D_ = 45.0 μM) than that of PPARγ LBD WT (Figure 5C,D), indicating that the PPARγ Q286E mutation attenuates the binding of endogenous ligands. When we compared the 15d-PGJ2- and 13S-HODE-bound PPARγ LBD structures (PDB ID: 2ZK1, 2VST) with our PPARγ Q286E mutant structure, the conformation of Glu286 could increase steric hindrance, reducing the binding affinities of endogenous ligands (Figure 5E). It was consistent with the decreased binding affinity of lobeglitazone for the PPARγ LBD Q286E mutant although PPARγ full agonist lobeglitazone still bound to the Q286E mutant with high affinity (Figure 2C). For the other helix H3 mutants, we also observed lower binding affinities and more steric clashes for 15d-PGJ2 and 13S-HODE than the PPARγ LBD WT (Figure 5F and Figure S9). Taken all together, although helix H3 mutations of PPARγ LBD found in cancers resulted in the attenuation of endogenous ligand binding due to steric hindrance, the PPARγ Q286E mutant could be potently activated regardless of ligand binding.

## 3. Discussion

In this study, we characterized helix H3 mutations of PPARγ LBD found in various cancers. We identified *PPARG* mutations from publicly available genomic databases or cancer-related publications and evaluated the structure–function relationship. We found that the PPARγ Q286E mutation confers ligand-independent PPARγ activation by stabilizing the constitutively active conformation of PPARγ, while PPARγ R280C, C285Y, Q286P, F287Y, R288C, R288H and S289C mutations are potential loss-of-function mutations in various aspects including ligand binding for PPARγ activation.

Although the exact roles of PPARγ in tumor microenvironments are still controversial, PPARγ is frequently regarded as a tumor suppressor, since numerous studies have shown that PPARγ transactivation by ligand binding induces inhibitory effects on tumor growth [12,13,14,15]. Considering the central role of helix H3 in ligand binding, PPARγ mutations in this region could potentially reduce PPARγ activation by attenuating ligand binding. However, the PPARγ Q286E mutation found in bladder cancer increased PPARγ transactivation by inducing the active conformation, favoring the recruitment of RXRα and co-activators without ligand binding (Figure 2, Figure 3, Figure 4 and Figure 5). It has recently been suggested that activation of the PPARγ/RXRα pathway via mutations in *PPARG* or *RXRA* promotes tumorigenesis in luminal bladder cancer [29,37]. The abnormal activation of the PPARγ/RXRα pathway decreases the activity of immune cell infiltration by inhibiting the expression and secretion of inflammatory factors, thereby hampering immunotherapies using immune checkpoint blockade in patients with cancer [30]. Indeed, several recurrent PPARγ mutations in luminal bladder cancer have been shown to induce the hyperactivation of PPARγ in a ligand-independent manner [25]. In a similar way, the PPARγ Q286E mutation in our study elicited transactivation regardless of ligand binding, thus supporting the pro-tumorigenic effects of PPARγ gain-of-function mutations in bladder cancer.

Transactivation by the PPARγ Q286E mutation could be explained by two structurally feasible mechanisms. First, the mutated Glu286 residue directly stabilizes the active conformation of PPARγ by forming a hydrogen bond with Tyr473 in helix H12 (Figure 2B). Full agonists bind to the ligand-binding pocket of PPARγ LBD, interact with Tyr473 via a hydrogen bond and stabilize helix H12 in the active conformation, which favors the recruitment of co-activators [38]. Similarly, the PPARγ Q286E mutation turns on the spontaneous recruitment of co-activators; it is worth noting that the PPARγ Q286E mutation is a distinct gain-of-function mutation as previously reported PPARγ recurrent T447M mutation (T475M in PPARγ isoform 2 numbering) directly stabilizing the active conformation of helix H12 [25]. Second, the structural stabilization of the Ω-loop, helix H3 and H11–H12 loop observed in the PPARγ Q286E structure would indirectly favor the active conformation (Figure 2D and Figure S5). In some cases, PPARγ ligands such as partial agonists do not directly interact with helix H12 but induce the transactivation of PPARγ [39,40,41,42]. These ligands have been suggested to indirectly elicit the transactivation of PPARγ by stabilizing the helix H3 and Ω-loop via an alternate binding site. Interestingly, our PPARγ Q286E structure in the absence of ligands exhibited the structurally stabilized helix H3 and Ω-loop because the Ω-loop forms extensive interaction networks with the helix H3 and H11–H12 loop (Figure 2D). When we calculated the normalized B-factors of the PPARγ LBD WT and Q286E structures, the regions encompassing Ω-loop, helix H3 and H11–H12 loop were more stable in the PPARγ LBD Q286E structure than in the PPARγ LBD WT structure (Figure S10). Thus, it would indirectly induce transactivation of the PPARγ Q286E mutant. Taken together, the novel structural aspects of the PPARγ LBD Q286E mutant could explain the PPARγ transactivation in a ligand-independent manner and the modulation of its pro-tumorigenic role in bladder cancer.

PPARγ mutations found in colon cancer have been reported to elicit various levels of PPARγ transcriptional activity [43]. However, our results showed that the helix H3 mutations of PPARγ LBD found in colon cancers, including PPARγ C285Y, Q286P, R288H and S289C mutations, exhibited lower binding affinities for endogenous ligands such as 15d-PGJ2 and 13S-HODE [35,36,44,45] (Figure 5F). Moreover, the PPARγ R288H mutation elicited lower binding affinity for RXRα and PPARγ Q286P and S289C mutations exhibited lower binding affinities for MED1 co-activator (Figure 3). Taken all together, the helix H3 mutations of PPARγ LBD found in colon cancers would be loss-of-function mutations that reduce transcriptional activity of PPARγ. In addition to colon cancer-associated mutations, PPARγ R280C and R288C found in uterine and skin cancers reduced the transcriptional activity of PPARγ and resulted in remarkably weak binding affinities for RXRα and MED1 co-activators (Figure 3 and Figure 4). PPARγ R280C and R288C mutations also seemed to inhibit the binding of endogenous ligands such as 15d-PGJ2 and 13S-HODE (Figure 5F). Collectively, the helix H3 mutations of PPARγ LBD found in various cancers disrupt the sophisticated ligand-binding systems of PPARγ, induce abnormal regulation of PPARγ transactivation and thus may affect tumor development or progression as loss-of-function mutations of PPARγ.

Recently, inhibition of PPARγ Ser245 phosphorylation by noncanonical agonist PPARγ ligands has been shown to deregulate tumor-suppressor p53 signaling, sensitizing cancer cells to cytotoxic chemotherapy [46]. Considering that phosphorylation levels of PPARγ Ser245 are regulated by various ligands, the reduced ligand binding from the PPARγ helix H3 mutations including Q286E might increase the phosphorylation level of PPARγ Ser245, resulting in cancer cell survival through the dysregulation of p53 signaling. Likewise, cancer-associated mutations in combination with various endogenous ligands might complicate PPARγ-dependent tumor microenvironment for different cancers, which should be further elucidated. In addition to PPARγ, the other PPAR subtypes PPARα and PPARδ have been also reported to play important roles in regulating cancer cell growth [47,48,49,50]. As a number of mutations in the other PPAR subtypes have been discovered in various cancers, our approach using structure-function analyses of cancer-associated mutations could be applied for the other PPAR subtypes, which will expand PPAR researches in the tumor microenvironment.

## 4. Materials and Methods 

### 4.1. Cloning, Expression and Mutagenesis

The cloning, expression, purification and crystallization of human PPARγ LBD were mainly performed as previously reported [39]. In brief, PPARγ LBD (residues 195–477 in PPARγ1 numbering) was cloned into the expression vector pET-28b(+) (Novagen, Darmstadt, Germany) between Nde1 and Xho1 restriction sites containing an N-terminal His_6_ tag (MGSSHHHHHHSSGLVPRGSH) and a thrombin cleavage site. The resulting recombinant PPARγ LBD proteins were overexpressed in *Escherichia coli* Rosetta 2(DE3) strain. Mutants of human PPARγ LBD in helix H3 region, including R280C, C285Y, Q286E, Q286P, F287Y, R288C, R288H and S289C, were generated by PCR (polymerase chain reaction)-based site-directed mutagenesis using PrimeSTAR^®^ HS DNA polymerase (Takara Bio Inc., Kusatsu, Japan) and the mutations were confirmed by DNA sequencing.

### 4.2. Purification

The cells containing recombinant PPARγ LBD WT or mutants were grown at 37 °C in Luria-Bertani broth media containing 30 μg/mL kanamycin and induced by 0.5 mM isopropyl 1-thio-β-D-galactopyranoside at an OD600 of 0.6 and then incubated for additional 18 h at 20 °C. The cells were harvested by centrifugation at 6000× *g* for 10 min and lysed by sonication in buffer A (20 mM Tris-HCl pH 8.5, 150 mM NaCl, 5 mM imidazole, 10% (*v/v*) glycerol, 1 mM TCEP) containing 1 mM phenylmethanesulfonylfluoride. The lysates were centrifuged at 35,000× *g* for an hour and the supernatants were filtered using a 0.45 μm syringe filter device (Sartorius, Göttingen, Germany). For an affinity chromatography, the supernatants were loaded onto 5 mL HiTrap chelating HP column (GE Healthcare, Chicago, IL, USA) that was pre-charged with Ni^2+^ and equilibrated with buffer A. Upon eluting with a linear gradient of buffer B (20 mM Tris-HCl pH 8.5, 150 mM NaCl, 300 mM imidazole, 10% (*v/v*) glycerol, 1 mM TCEP), PPARγ LBD proteins were eluted at 50–100 mM imidazole concentrations. After the eluted proteins were desalted using HiPrep Desalting column 26/10 (GE Healthcare) to buffer C (20 mM Tris-HCl pH 8.5, 150 mM NaCl, 10% (*v/v*) glycerol, 1 mM TCEP), the proteins were treated with thrombin (Sigma-Aldrich, Darmstadt, Germany) at 1 unit/mg and incubated at 4 °C overnight for the cleavage of His_6_-tags. The His_6_-tag-cleaved PPARγ LBD proteins were purified from the His_6_-tags by passing through the Ni^2+^ charged HiTrap chelating HP column and applied on a gel filtration chromatography column of HiLoad 16/600 Superdex200 pg (GE Healthcare) which was previously equilibrated with buffer C. For crystallization, the purified PPARγ LBD proteins were concentrated to 15.5 mg/mL using an Amicon Ultra-15 Centrifugal Filter Unit (Merck Millipore, Darmstadt, Germany). The recombinant PPARγ LBD mutants of R280C, C285Y, Q286E, Q286P, F287Y, R288C, R288H and S289C were expressed and purified in the same manner as the WT protein.

### 4.3. Crystallization and X-Ray Data Collection

The crystals of PPARγ LBD mutants in the presence of SRC-1 peptide (ERHKILHRLLQEGSPS) were grown by the sitting-drop vapor diffusion method at 22 °C by mixing equal volumes (0.5 μL) of PPARγ LBD protein and crystallization solution containing 2.2 M sodium malonate pH 7.0. The crystals of PPARγ LBD Q286E and R280C in the absence of a SRC-1 peptide were grown by mixing equal volumes (0.5 μL) of protein and crystallization solution containing 1.4 M sodium citrate tribasic dihydrate (Hampton Research, Aliso Viejo, CA, USA) and 0.1 M HEPES pH 7.5. Crystals that are suitable for data collection were grown in the presence of micro-seeds that were made from initial crystals of PPARγ LBD WT using Seed Bead^TM^ (Hampton Research) according to the manufacturer’s instructions. X-ray diffraction data for PPARγ LBD mutants were collected at 100 K in BL-5C and BL-7A experimental stations at Pohang Light Source, Korea and in PF-1A and PF-NE3A experimental stations at Photon Factory, Japan. All data were processed and scaled using *HKL*2000 [51]. Crystals of PPARγ LBD mutants in the presence of SRC-1 peptide belong to the space group of *P*2_1_2_1_2 and crystals of PPARγ LBD mutants in the absence of SRC-1 peptide belong to the space group *C*2, with similar cell parameters of PPARγ LBD WT. Data collection statistics are summarized in Table S1.

### 4.4. Structure Determination and Refinement

The mutant structures were determined by molecular replacement with the previously published ligand-free PPARγ LBD WT structure models (PDB ID: 5GTP for peptide-bound structures; PDB ID: 6L8B for peptide-unbound structures) using MolRep [52]. The structures were refined by iterative manual buildings using Coot [53] and Refmac5 [54] in the CCP4 program suite. All refinement steps were monitored using R_free_ –values [55] based on excluded reflections, and reliabilities of refined models were evaluated using MolProbity [56]. The statistics for refinements are summarized in Table S1.

### 4.5. Surface Plasmon Resonance

The binding affinities of PPARγ LBD with lobeglitazone, RXRα and MED1 were investigated by SPR kinetics experiments. All SPR experiments were conducted at 25 °C in a Biacore T200 system (GE Healthcare). For immobilization, an amine coupling kit containing 0.1 M N-hydroxysuccinimide and 0.4 M 1-ethyl-3-(3-dimethylaminopropyl) carbodiimide hydrochloride on a CM5 sensor chip with HBS-EP (HEPES-buffered saline with EDTA and surfactant P20) buffer (10 mM HEPES pH 7.5, 150 mM NaCl, 3 mM EDTA, 0.005% tween 20) was used according to the manufacturer’s protocol (GE Healthcare). For binding affinity measurements between PPARγ LBD and lobeglitazone, 30 μg/mL PPARγ LBD WT or Q286E mutant dissolved in 10 mM sodium acetate at pH 5.0 were injected until the immobilization level reached approximately 3500 response units (RU) on the flow cell 2 or 4, respectively. The remaining activated carboxyl groups on the CM5 sensor chip surface were deactivated with 1 M ethanolamine at pH 8.5 for 400 s. The control experiment was treated identically with reference flow cell 1 or 3 without proteins and the response by the control was subtracted from each sample dataset. Lobeglitazone at concentrations of 78.12 nM, 156.25 nM, 312.5 nM, 625 nM and 1.25 μM in PBS (phosphate-buffered saline) buffer containing 5% (*v/v*) DMSO and 0.005% (*v/v*) Tween 20 was injected over the flow cell 1 to 4 at a rate of 30 μL/min for 120 s, followed by dissociation for 600 s in multi-cycle reactions. The sensor chip surface was regenerated for 5 s with 5 mM NaOH between cycles. For binding affinity measurements between PPARγ LBD and RXRα LBD, 5 μg/mL RXRα LBD dissolved in 10 mM sodium acetate at pH 5.0 were injected until the immobilization level reached approximately 600 RU. PPARγ LBD WT and mutants (R280C, C285Y, Q286E, Q286P, F287Y, R288C, R288H and S289C) at concentrations of 3.125 nM, 6.25 nM, 12.5 nM, 25 nM, 50 nM, 100 nM and 200 nM in the buffer of 50 mM Tris-HCl at pH 7.5, 150 mM NaCl, 1 mM TCEP, 0.005% (*v/v*) tween 20 were injected over the chip at a rate of 30 μL/min for 120 s, followed by dissociation for 500 s in multi-cycle reactions without regenerations between cycles. For binding affinity measurements between PPARγ LBD and MED1 peptide, 100 μg/mL MED1 peptide dissolved in 10 mM sodium acetate at pH 5.5 were injected until the immobilization level reached approximately 60 RU. PPARγ LBD WT and mutants (R280C, C285Y, Q286E, Q286P, F287Y, R288C, R288H and S289C) at concentrations of 78 nM, 156 nM, 312 nM, 625 nM, 1.25 μM, 2.5 μM, 5 μM and 10 μM in the buffer of 50 mM Tris-HCl at pH 7.5, 150 mM NaCl, 1 mM TCEP, 0.005% (*v/v*) tween 20 were injected over the chip at a rate of 30 μL/min for 120 s, followed by dissociation for 400 s in multi-cycle reactions without regenerations between cycles. The SPR response data were fit to the simple bimolecular 1:1 Langmuir isotherm binding model to determine the equilibrium dissociation constant (*K*_D_) using Biacore T200 evaluation software 3.0 (GE Healthcare).

### 4.6. Luciferase Reporter Gene Assay

HEK293T cells were cultured in Dulbecco Modified Eagles’ Medium (DMEM; glucose 1 g/L) supplemented with 10% fetal bovine serum and 1% (*v/v*) penicillin and streptomycin. Cells were grown to 80% confluency at 37 °C under an atmosphere containing 5% CO_2_ and then plated in 96-well plates (10,000 cells per well). For luciferase assays, HEK293T cells were transfected with 50 ng pcDNA3-PPARγ WT or mutants (R280C, C285Y, Q286E, Q286P, F287Y, R288C, R288H and S289C), 70 ng PPRE-X3-TK-luc (1015; Bruce Spiegelman, Addgene, Cambridge, MA, USA) and 10 ng pRL-SV40 (Promega, Wallisellen, Switzerland) using Lipofectamine 2000 (Invitrogen, Carlsbad, CA) in opti-MEM according to the manufacturer’s instruction. The media were changed to the culture media after 4 h and the cells were incubated for additional 48 h. Luciferase activity was determined using Dual-Glo^®^ Luciferase Assay System 2000 (Promega), according to the manufacturer’s instruction and the resulting signals were normalized with *Renilla* luciferase signals obtained from pRL-SV40 plasmids. Relative luciferase units were plotted.

### 4.7. Total RNA Isolation and Quantitative Real-Time PCR

The total RNA samples in HEK293T cells containing the expression vectors for PPARγ WT and Q286E mutant were isolated using TRIzol (Thermo Fisher Scientific, Waltham, MA, USA). Reverse transcription was conducted with 1 μg of total RNA using Moloney Murine Leukemia Virus (M-MLV) Reverse Transcriptase (Promega). Real-time quantitative PCR was performed with an Applied Biosystems 7500 Fast Real-Time PCR Instrument System (Thermo Fisher Scientific) using RealHelix Premier Quantitative PCR Kit (NanoHelix Co. Ltd., Deajeon, Korea) and primer pairs as follows: PLIN2: forward primer-TGTTAACAACACGCCCCTCA; reverse primer-ATGCTCAGATCGCTGGGGTCT; ACSL5: forward primer-TAGCTTCGGGTGTGTCCCAG; reverse primer-CCCTCACAGCTGGCTGTATT; FABP4: forward primer-TGGGCCAGGAATTTGACGAA; reverse primer-CACATGTACCAGGACACCCC. The identity of PCR products was verified using the dissociation curve analysis. Target gene expression was normalized to the gene of human *L32*. Data were analyzed by the comparative cycle threshold (∆∆C_t_) method.

### 4.8. Isothermal Titration Calorimetry

Binding affinities of PPARγ LBD WT and mutants (R280C, C285Y, Q286E, Q286P, F287Y, R288C, R288H and S289C) for 15-deoxy-∆^12,14^-prostaglandin J_2_ (15d-PGJ2, Sigma Aldrich) and 13S-hydroxy-9Z,11E-octadecadienoic acid (13S-HODE, Cayman Chemical, Ann Arbor, MI, USA) were measured using a MicroCal ITC200 system (Malvern Instruments, Worcestershire, UK). All the measurements were conducted in the buffer of PBS pH 7.2 at 25 °C. The ligands filled in the syringe were titrated into the proteins in the cell using 19 injections of 2 μL and an initial injection of 0.4 μL with 150 s equilibration between injections. The final protein concentrations of PPARγ LBD WT and mutants were 60 μM for 15d-PGJ2 at the ligand concentration of 900 μM and 45 μM for 13S-HODE at the ligand concentration of 900 μM, respectively. The obtained data were analyzed using Origin 7.0 software.

### 4.9. Data Availability

Atomic coordinates and structure factors for the structures of PPARγ LBD C285Y, Q286E, F287Y, R288C, R288H and S289C in presence of SRC-1 peptide and the structures of PPARγ LBD R280C and Q286E in the absence of SRC-1 peptide have been deposited in Protein Data Bank under the accession codes 7CXF, 7CXH, 7CXI, 7CXJ, 7CXK, 7CXL, 7CXE and 7CXG, respectively.

The mutation datasets in this study are available in The Cancer Genome Atlas (http://cancergenome.nih.gov), International Cancer Genome Consortium (http://dcc.icgc.org), cBioPortal (http://www.cbioportal.org), and Catalogue Of Somatic Mutations In Cancer (http://cancer.sanger.ac.uk/cosmic) servers.

## 5. Conclusions

In the context of the rapid accumulation of cancer-related information about genetic alterations in *PPARG*, our systematic analyses on the structure–function relationships of cancer-associated PPARγ mutations enriched in helix H3 would provide a quantitative approach to predict cancer development and progression. In addition to the representative PPARγ research methods adopted in this study, further investigations of the physiological mechanisms by which PPARγ mutations influence the tumor microenvironment are needed. Our extensive characterization of cancer-associated mutations in helix H3 of PPARγ LBD provides a stepping stone for the development of new biomarkers targeting tumor microenvironments and a new therapeutic approach that pharmacologically inhibits the abnormal activation of PPARγ/RXRα pathway observed in certain cancers.

## Supplementary Materials

The following are available online at https://www.mdpi.com/2072-6694/12/12/3580/s1, Figure S1: Magnified images of the mutated residues from the PPARγ LBD helix H3 mutants, Figure S2: Overall structures of PPARγ LBD WT and mutants, Figure S3: CD spectra of PPARγ LBD WT, Q286E and Q286P, Figure S4: Overall structures of PPARγ LBD WT and Q286E in the absence of a SRC-1 peptide, Figure S5: Close-up view of the interaction network among helix H3, Ω-loop and H11-H12 loop in the structure of PPARγ LBD Q286E, Figure S6: Structural comparison of PPARγ LBD WT (PDB ID: 6L8B) (colored in white) and Q286E (colored in salmon) in the absence of a SRC-1 peptide, Figure S7: Sensorgrams for the summary (Figure 3C) showing the binding affinity between RXRα LBD and PPARγ LBD, Figure S8: Sensorgrams for the summary (Figure 3F) showing the binding affinity between a MED1 peptide and PPARγ LBD, Figure S9: Superposition of the mutated residues in PPARγ LBD helix H3 and known PPARγ endogenous ligands from reported complex structures, Figure S10: Normalized B-factor comparison between PPARγ LBD WT and Q286E structures, Table S1: Statistics for the data collection and model refinement, Table S2: Cancer-associated helix H3 mutations of PPARγ LBD.
