# Supplementary material for: Differential Effects of Cancer-Associated Mutations Enriched in Helix H3 of PPARγ
Source: Cancers (Basel). 2020 Nov 30;12(12):3580. doi: 10.3390/cancers12123580 (PMC7761077; doi:10.3390/cancers12123580)
Supplement: Supplementary file 1 [file cancers-12-03580-s001.pdf]

cancers

Supplementary Materials:

Differential effects of cancer-associated mutations enriched in helix H3 of PPARγ

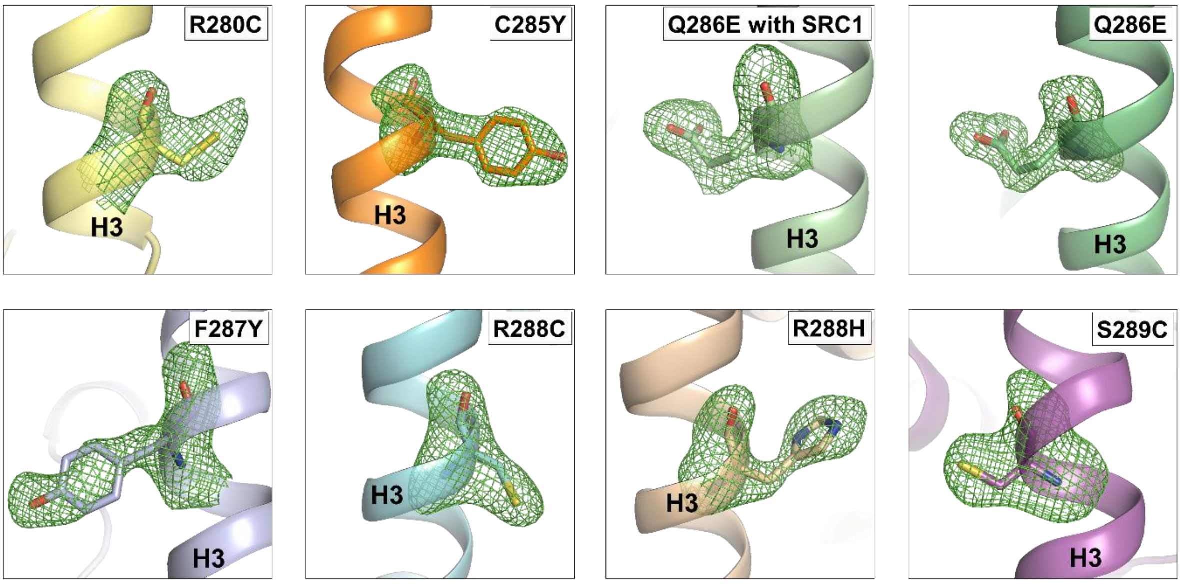

Figure S1. Magnified images of the mutated residues from the PPARγ LBD helix H3 mutants. The mutated residues are shown as stick representations with the m*Fo*-*DF*c electron density omit maps (contoured at 2.5σ), which are displayed as green-colored meshes.

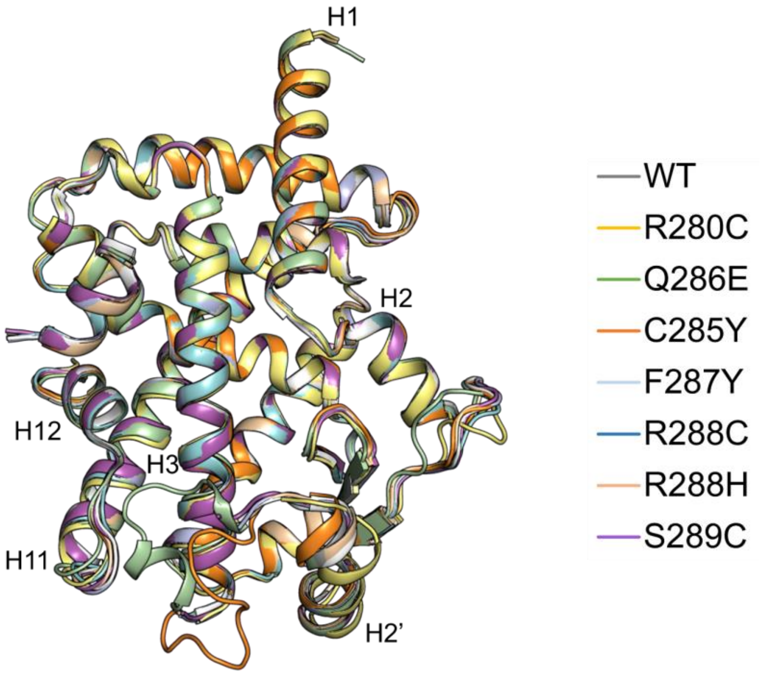

Figure S2. Overall structures of PPARγ LBD WT and mutants. The PPARγ LBD mutant structures are superposed to the PPARγ LBD WT structure (PDB ID: 5GTP) and shown as cartoon representations colored as depicted on the right side.

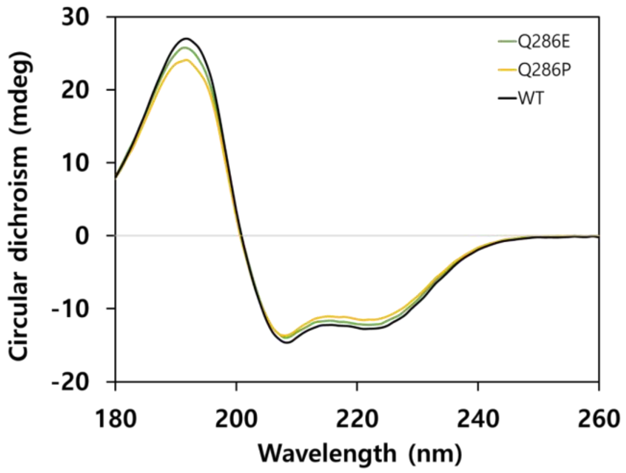

PPARγ \ SS	α-helix	β-sheet	turn	random
WT	42.8	9.6	12.5	35.1
Q286E	42.5	10.2	12.5	34.8
Q286P	40.8	10.2	12.5	36.5

(%)

Figure S3. CD spectra of PPARγ LBD WT, Q286E, and Q286P. The secondary structure contents (%) are calculated from the CD spectra results and summarized in the table below. CD spectroscopy is performed with the Chiranscan™ –plus CD spectrometer (Applied photophysics Ltd., Surrey, UK) at 298 K with a wavelength range from 260 nm to 180 nM. The proteins of PPARγ LBD WT, Q286E, and Q286P at a concentration of 0.4 mg/mL in a buffer containing 50 mM sodium fluoride and 10 mM potassium phosphate dibasic pH 7.5 have 0.8 maximum absorbance in a cuvette of 1.5 mm bandwidth at the temperature of 298 K.

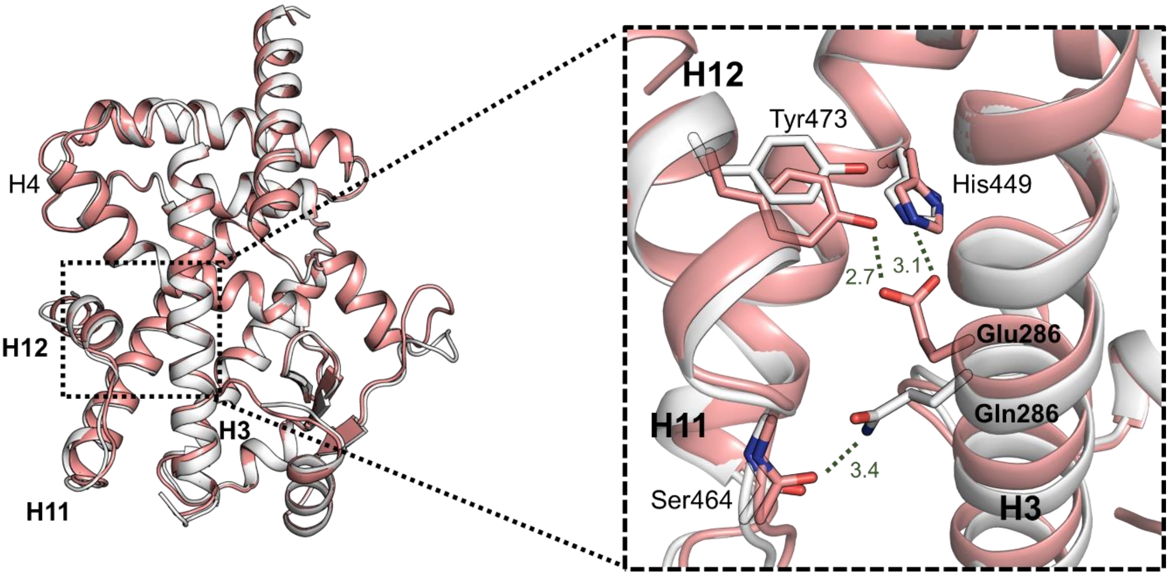

Figure S4. Overall structures of PPARγ LBD WT and Q286E in the absence of a SRC-1 peptide. Superposition of PPARγ LBD WT (white) and Q286E (salmon) structures. Overall structures are displayed as cartoon representations. The region of helices H3, H11, and H12 is magnified and depicted by a black-dashed box. Stick representations are shown for the backbone of Ser464 and the

side chains of Tyr473, His449, Glu286, and Gln286. Hydrogen bonds are depicted by green dashed lines and labeled with distances in Å.

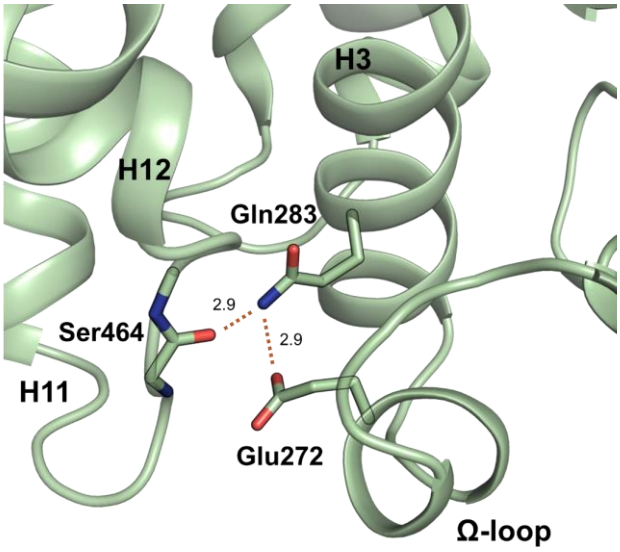

Figure S5. Close-up view of the interaction network among helix H3, Ω-loop, and H11-H12 loop in the structure of PPARγ LBD Q286E. The side chains of Gln283 and Glu272 and the backbone of Ser464 are shown as a stick representation. The hydrogen bonds are depicted as dashed lines with distances in Å.

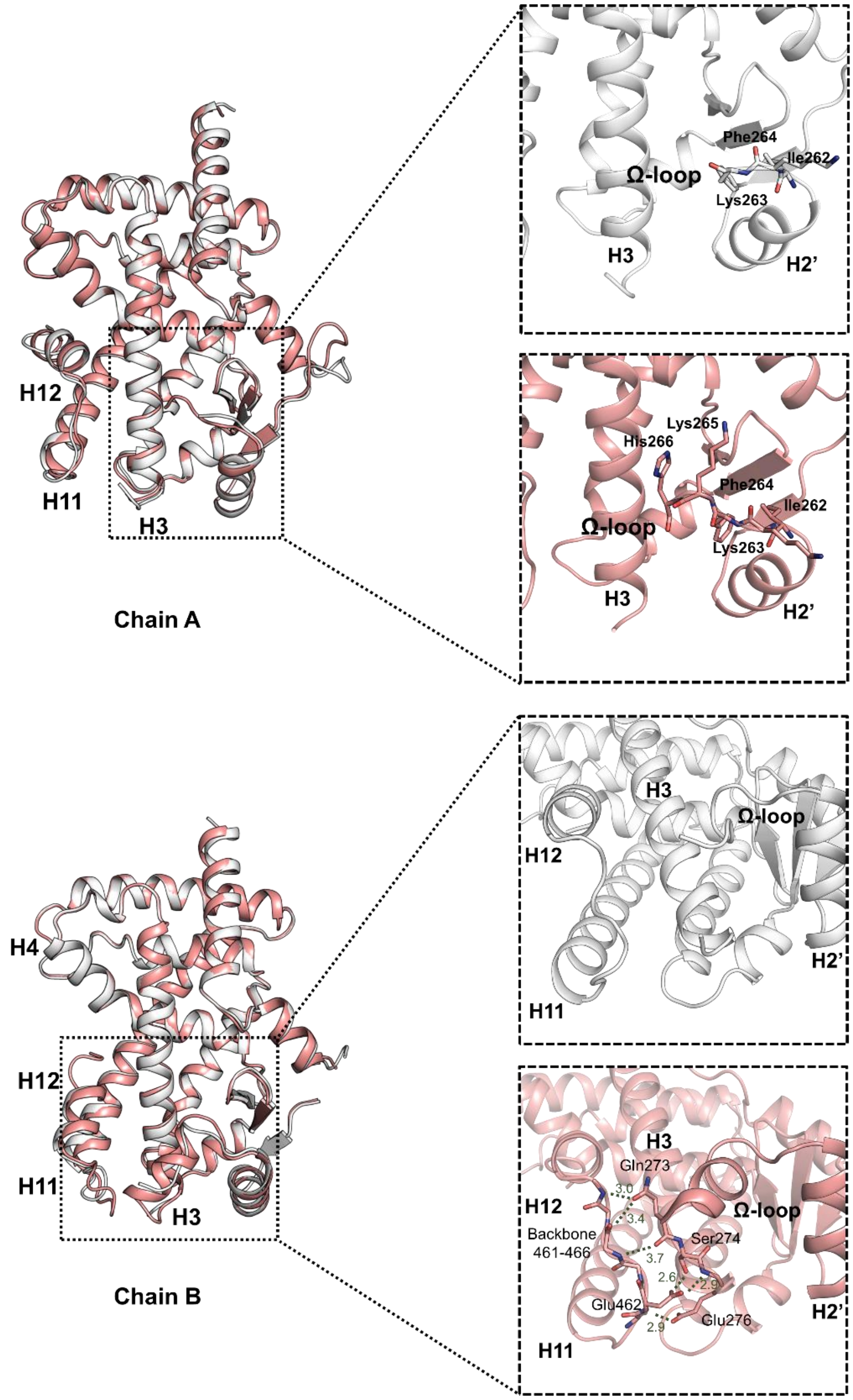

Figure S6. Structural comparison of PPARγ LBD WT (PDB ID: 6L8B) (colored in white) and Q286E (colored in salmon) in the absence of a SRC-1 peptide. The structures of PPARγ LBD WT and Q286E are superposed in chain A (above) and chain B (below). The region of Ω-loop is magnified and depicted by a black-dashed box. Hydrogen bonds are depicted by green dashed lines and labeled with distances in Å. Oxygen and nitrogen atoms are colored in red and blue, respectively.

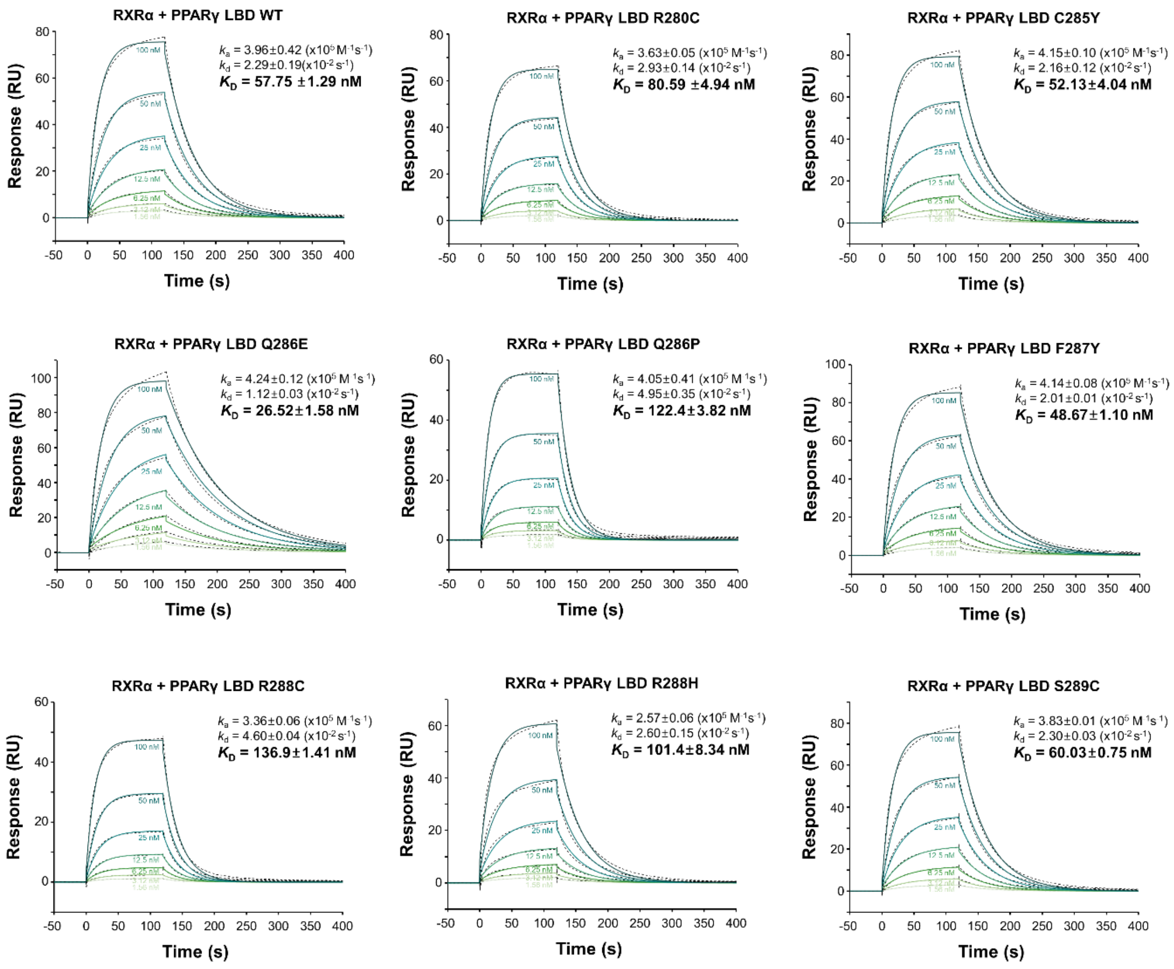

Figure S7. Sensorgrams for the summary (Figure 3C) showing the binding affinity between RXR*α* LBD and PPARγ LBD. The dissociation constants of PPARγ LBD WT and mutants for RXR*α* were calculated from the kinetic measurement. The data of k_a, k_d, and K_D are presented as the mean ± SD of two independent experiments.

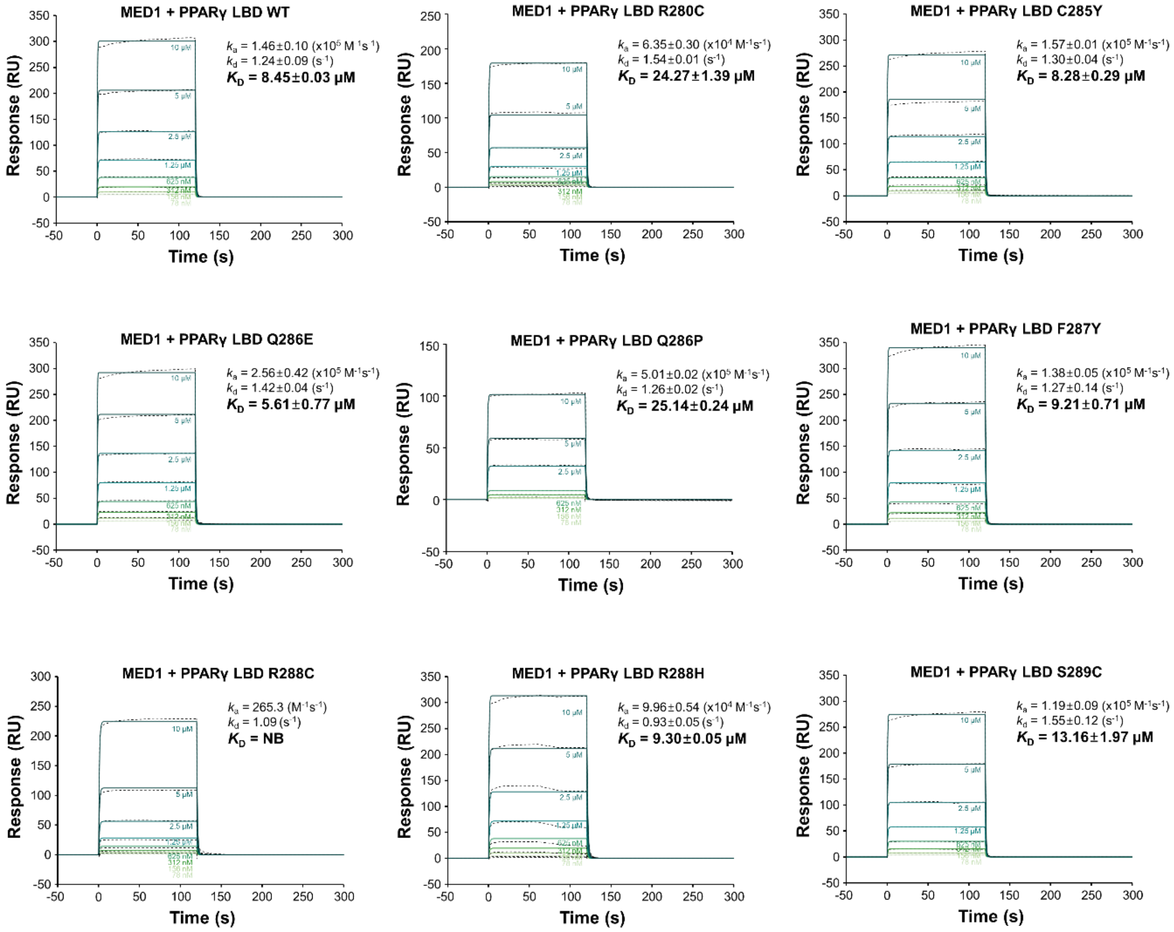

Figure S8. Sensorgrams for the summary (Figure 3F) showing the binding affinity between a MED1 peptide and PPARγ LBD. The dissociation constants of PPARγ LBD WT and mutants for RXR*α* were calculated from the kinetic measurement. The data of k_a, k_d, and K_D are presented as the mean ± SD of two independent experiments.

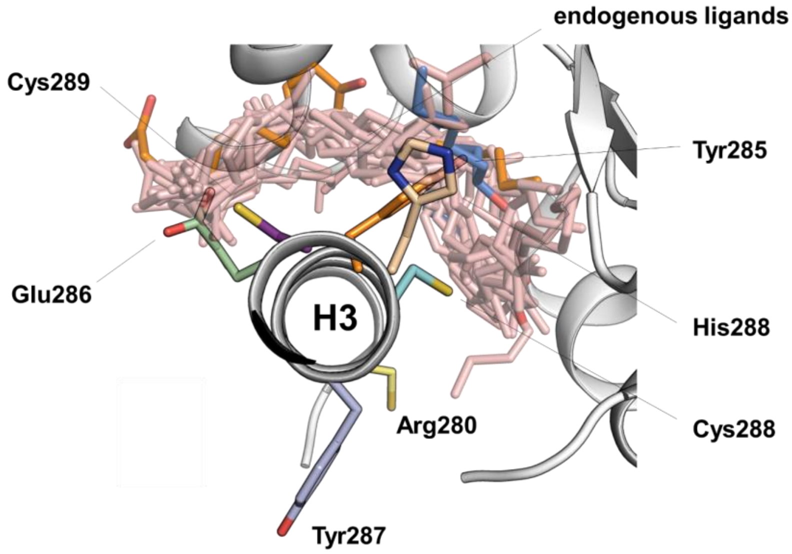

Figure S9. Superposition of the mutated residues in PPARγ LBD helix H3 and known PPARγ endogenous ligands from reported complex structures. In total, 20 ligand-bound PPARγ LBD structures in the protein data bank were superposed onto the PPARγ LBD WT structure (cartoon in white color). The ligands are displayed as stick representations colored in pink. The PPARγ LBD

mutant structures (R280C, C285Y, Q286E, F287Y, R288C, R288H, and S289C) were superposed onto the PPARγ LBD WT structure. The mutant residues are shown as stick representations in various colors.

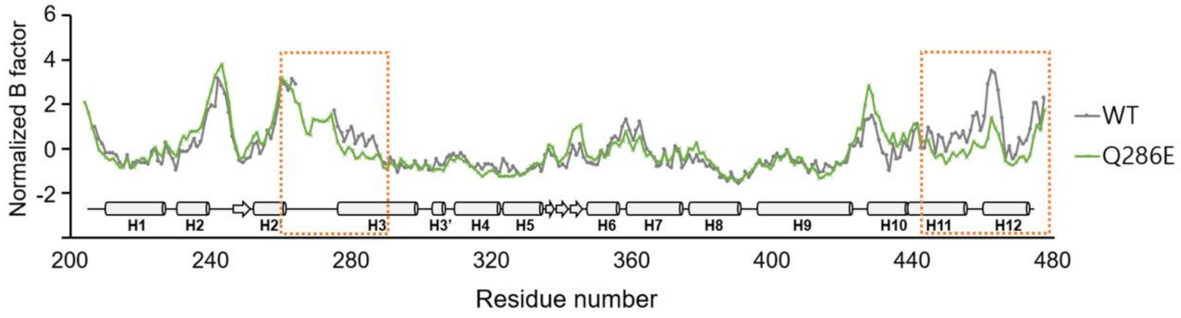

Figure S10. Normalized B-factor comparison between PPARγ LBD WT and Q286E structures. The helices H3, H11, and H12 in the PPARγ LBD Q286E structure show lower B-factor values than those in the PPARγ LBD WT structure indicated by orange dashed rectangles. Secondary structure elements are indicated on the residue number.

Table S1-1. Statistics for the data collection and model refinement.

	**PPARγ LBD R280C**	**PPARγ LBD C285Y with a SRC-1**	**PPARγ LBD Q286E**	**PPARγ LBD Q286E with a SRC-1**
**PDB ID**	**7CXE**	**7CXF**	**7CXG**	**7CXH**
**Data collection**				
**X-ray source**	PF-1A	PLS-7A	PF-NE3A	PLS-7A
**X-ray wavelength (Å)**	1.1000	0.97940	1.0000	0.97934
**Space group**	*C*2	$P2_12_12$	*C*2	$P2_12_12$
**Cell dimensions**				
**a, b, c (Å)**	93.94, 61.28, 119.50	130.73, 51.94, 54.13	91.99, 59.59, 117.76	129.44, 52.13, 54.42
**α, β, γ (°)**	90.00, 102.42, 90.00	90.00, 90.00, 90.00	90.00, 103.40, 90.00	90.00, 90.00, 90.00
**Resolution range (Å)**	50.00-2.50 (2.54-2.50)[a]	50-2.35 (2.39-2.35)[a]	50.00-1.88 (1.91-1.88)[a]	50.00–2.25 (2.29-2.25)[a]
**Total/unique reflections**	110,753/21,448	91,866/16,225	265,752/51,089	145,603/18,418
**Redundancy**	5.2 (5.3)[a]	5.7 (5.8)[a]	5.2 (5.0)[a]	7.9 (8.1)[a]
**Completeness (%)**	91.0 (89.1)[a]	99.0 (97.9)[a]	99.9 (99.7)[a]	100 (100)[a]
**$\langle I/\sigma_I \rangle$**	19.43 (2.06)[a]	28.21 (2.64)[a]	22.49 (1.96)[a]	19.28 (2.81)[a]
**R_{merge}[b] (%)**	7.2 (58.4)[a]	5.3 (58.4)[a]	6.5 (69.7)[a]	10.6 (72.9)[a]
**$CC_{1/2}$**	100 (87.5)[a]	100 (87.4)[a]	99.0 (75.1)[a]	98.5 (86.6)[a]
**Model refinement**				
**Resolution range (Å)**	50.00-2.50	50.00-2.35	50.00-1.88	50.00-2.30
**R_{work}/R_{free}[c] (%)**	21.56/26.45	22.92/26.93	18.66/23.08	21.20/25.70
**No. of non-hydrogen atoms**				
**Protein**	4137	2291	4257	2301
**Ligand/ion**	-	7 (malonate)	17 (glycerol)	-
**Water**	50	61	297	90
**Average B-factors**				
**Protein**	44.57	41.02	22.87	37.14
**Ligand/ion**	-	48.07 (malonate)	43.18 (glycerol)	-

**Water**	34.34	41.65	30.06	34.71
**R.m.s. deviations**				
**Bond lengths (Å)**	0.0059	0.0044	0.0044	0.0041
**Bond angles (°)**	1.2157	1.2687	1.2263	1.2613
**Ramachandran plot[d]**				
**Favored / Outliers (%)**	97.63/0.00	96.77/0.00	99.04/0.00	98.58/0.00
**Rotamer outliers[d] (%)**	0.00	0.00	0.00	0.00

[a] Values in parentheses refer to the highest resolution shell. [b] $R_{merge} = \Sigma_h\Sigma_i|I(h)_i - \langle I(h)\rangle|/\Sigma_h\Sigma_i I(h)_i$, where I(h) is the intensity of reflection h, Σ_h is the sum over all reflections, and Σ_i is the sum over i measurements of reflection h. [C] $R_{free} = \Sigma||F_{obs}|-|F_{calc}||/\Sigma|F_{obs}|$, where R_{free} is calculated for a randomly chosen 5% of reflections that are not used for structure refinement. R_{work} is calculated for the remaining reflections. [d] Values are obtained using *MolProbity*.

Table S1-2. Statistics for the data collection and model refinement.

	**PPARγ LBD F287Y with a SRC-1**	**PPARγ LBD R288C with a SRC-1**	**PPARγ LBD R288H with a SRC-1**	**PPARγ LBD S289C with a SRC-1**
**PDB ID**	**7CXI**	**7CXJ**	**7CXK**	**7CXL**
**Data collection**				
**X-ray source**	PLS-5C	PLS-7A	PLS-7A	PLS-7A
**X-ray wavelength (Å)**	0.97940	0.97934	0.9793	0.97934
**Space group**	$P2_12_12$	$P2_12_12$	$P2_12_12$	$P2_12_12$
**Cell dimensions**				
**a, b, c (Å)**	130.75, 52.38, 53.97	131.25, 52.88, 53.89	130.40, 52.00, 54.05	130.87, 53.16, 53.68
**α, β, γ (°)**	90.00, 90.00, 90.00	90.00, 90.00, 90.00	90.00, 90.00, 90.00	90.00, 90.00, 90.00
**Resolution range (Å)**	50.00–2.30 (2.34-2.30)[a]	50.00–2.65 (2.70-2.65)[a]	50.00–2.25 (2.24-2.20)[a]	50.00–2.70 (2.75-2.70)[a]
**Total/unique reflections**	131898/17093	89344/11616	190289/19382	84511/10850
**Redundancy**	7.7 (7.8)[a]	7.7 (7.8)[a]	9.8 (10.1)[a]	7.8 (8.2)[a]
**Completeness (%)**	99.6 (100)[a]	99.6 (99.1)[a]	99.7 (100)[a]	100 (100)[a]
**$\langle I/\sigma_I \rangle$**	31.8 (3.06)[a]	28.03 (2.69)[a]	31.63 (4.31)[a]	25.16 (4.00)[a]
**R_{merge}[b] (%)**	5.4 (66.0)[a]	6.9 (75.9)[a]	7.3 (64.6)[a]	8.0 (60.8)[a]
**$CC_{1/2}$**	99.4 (88.4)[a]	99.7 (87.3)[a]	99.5 (92.0)[a]	99.4 (88.0)[a]
**Model refinement**				
**Resolution range (Å)**	50.00-2.30	50.00-2.65	50.00-2.25	30.00-2.70
**R_{work}/R_{free}[c] (%)**	24.34/27.61	23.96/27.36	23.69/26.36	23.81/25.50
**No. of non-hydrogen atoms**				
**Protein**	2197	2168	2219	2205
**Ligand/ion**	7 (malonate)	-	7 (malonate)	7 (malonate)
**Water**	26	19	77	62
**Average B-factors**				
**Protein**	53.30	43.00	41.15	46.08
**Ligand/ion**	63.35 (malonate)	-	59.45 (malonate)	86.59 (malonate)
**Water**	41.59	33.10	37.67	34.55
**R.m.s. deviations**				
**Bond lengths (Å)**	0.0048	0.0057	0.0091	0.0095
**Bond angles (°)**	1.3065	1.2419	1.3649	1.3048

**Ramachandran plot**^{d}				
**Favored / Outliers (%)**	96.24/0.00	96.59/0.00	98.14/0.00	98.50/0.00
**Rotamer outliers**^{d} **(%)**	0.00	0.00	0.00	0.00

^{a} Values in parentheses refer to the highest resolution shell. ^{b} $R_{merge} = \Sigma_h\Sigma_i|I(h)_i - \langle I(h)\rangle|/\Sigma_h\Sigma_i I(h)_i$, where I(h) is the intensity of reflection h, Σ_h is the sum over all reflections, and Σ_i is the sum over i measurements of reflection h. ^{C} $R_{free} = \Sigma||F_{obs}| - |F_{calc}||/\Sigma|F_{obs}|$, where R_{free} is calculated for a randomly chosen 5% of reflections that are not used for structure refinement. R_{work} is calculated for the remaining reflections. ^{d} Values are obtained using *MolProbity*.

Table S2. Cancer-associated helix H3 mutations of PPARγ LBD.

AA change	Nucleotide change	Cancer_Type	Mutation type	Sample type	Sample ID	Source
R280C (R308C)	838C>T (922C>T)	Central nervous system; Brain	missense_mutation	patient	MB65	ICGC
		Endometrium (Endometrioid carcinoma)			BS-A0UV-01	TCGA
C285Y (C313Y)	854G>A (938G>A)	Colorectal Large intestine	missense_mutation	patient	T3563	DFCI 2016
Q286E (Q314E)	856C>G (940C>G)	Bladder	missense_mutation	patient	B88	BGI 2013
					DO48444	ICGC
Q286P (Q314P)	857A>G (941A>G)	Colorectal	missense_mutation	patient	HC-784	publications
F287Y (F315Y)	860T>A (944T>A)	Cutaneous squamous cell carcinoma (skin)	missense_mutation	patient	S10-24679-TP	DFCI 2015
R288C (R316C)	862C>T (946C>T)	Skin (Malignant melanoma)	missense_mutation	patient	D3-A1Q4-06	TCGA
R288H (R316H)	863G>A (947G>A)	Colorectal	missense_mutation	patient	HC-840	publications
S289C (S317C)	866C>G (950C>G)	Colorectal	missense_mutation	patient	-	publications
